# Crew resource management in the ICU: the need for culture change

**DOI:** 10.1186/2110-5820-2-39

**Published:** 2012-08-22

**Authors:** Marck HTM Haerkens, Donald H Jenkins, Johannes G van der Hoeven

**Affiliations:** 1 Department of Intensive Care Medicine, Radboud University Nijmegen Medical Centre, Braspenninglaan 2, 5337, NK ‘s-Hertogenbosch, The Netherlands; 2 Division of Trauma and Critical Care, Mayo Clinic, Rochester, MN, USA; 3 Department of Intensive Care Medicine, Radboud University Nijmegen Medical Centre, Nijmegen, The Netherlands

**Keywords:** Intensive care, Human factors, Safety climate, Crew resource management

## Abstract

Intensive care frequently results in unintentional harm to patients and statistics don’t seem to improve. The ICU environment is especially unforgiving for mistakes due to the multidisciplinary, time-critical nature of care and vulnerability of the patients. Human factors account for the majority of adverse events and a sound safety climate is therefore essential. This article reviews the existing literature on aviation-derived training called Crew Resource Management (CRM) and discusses its application in critical care medicine. CRM focuses on teamwork, threat and error management and blame free discussion of human mistakes. Though evidence is still scarce, the authors consider CRM to be a promising tool for culture change in the ICU setting, if supported by leadership and well-designed follow-up.

## Introduction

Despite modern equipment, continuing research and excellent training facilities our western health care system has a serious safety problem. It is estimated that out of all hospital admissions 2.9% to 16.6% suffer unintentional harm and in the USA alone up to 100,000 people may die as a result of medical errors [1]. Data from the Netherlands (2004) seem to support this with an annual number of 30.000 patients suffering preventable harm including approximately 1.735 avoidable deaths [2]. The financial cost of avoidable adverse events was estimated to be 1% of the hospital total budget [3].

These alarming reports resulted internationally in increased pressure to improve patient safety. In line with the current safety paradigm that limiting human variability in otherwise safe systems will lead to safer care [4], this resulted in more stringent procedural guidelines. Checklists, time-outs and safety management systems were subsequently implemented [5]. Unfortunately, current efforts have not eliminated human error [2] and as expected matters are worse in the ICU environment [6-8]. Patients in the ICU frequently suffer from severe, multiple-system disorders that require more testing, monitoring, invasive treatment and intravenous medications than in any other hospital department [9]. Errors in this setting have a greater impact due to the increased vulnerability of the patient. The sheer number of available data essential to make a single decision is daunting. Furthermore, ‘rogue’ data (irrelevant but abnormal e.g. a high glucose value) unrelated to the true problem can cause a change or loss in focus on the ‘real’ problem. This puts great pressure on multidisciplinary planning and decision making and combined with the time-critical aspects of the ICU environment increases patient risk.

Identifying the key factors in safe critical care is a challenging task. Human factors appear to play an important role [10]. Less often organizational and technical factors are involved. This is consistent with publications from other critical industries [11].

### Safety climate

If safe critical care relies on the interdisciplinary performance of a care team as much as on individual expertise, it makes sense to establish a sound safety culture as the basis of improving patient outcomes [12]. From an anthropological standpoint, “safety culture” is only measurable by careful, long-term observations. Therefore, in the evidence –driven medical world it may be more appropriate to use the term “safety climate”, which can be assessed by questionnaires. The Safety Attitudes Questionnaire (SAQ) is widely regarded to be valid, reliable, psychometrically sound and responsive to interventions [13]. Evidence from other critical industries suggests that “safety climate” correlates with unsafe and safety-specific behavior, injury rates and accidents [14,15]. Likewise, in the medical literature the “safety climate” of a hospital unit is considered one of the main contributing factors to a better quality of care [16]. How could we improve this safety climate?

The majority of current interventions focus on implementing safety tools such as event-reporting systems, quality and safety dashboards, evidence-based guidelines and checklists [4]. Even though the results of a comprehensive unit-based safety program (CUSP) are promising [13,16], introducing more stringent rules potentially increases the gap between procedure and practice [17]. Therefore, the question remains if these tools can be truly effective in the traditional hospital climate, where highly trained professionals tend to focus more on individual performance than team effectiveness [18]. Moreover, the typical culture in which junior members of the ICU staff should not question the decisions made by senior members adds to the challenge [19].

### Parallels

We can definitely learn from methods developed in other critical industries, despite the perceived procedural differences with health care [20,21]. One example is the professional civilian or military aviation industry. Up to 1977 aviation industry could be classified as a professional-centered, hierarchic working environment. This all changed with the Tenerife disaster. A KLM Boeing 747 at take-off crashed into Pan Am flight 736 still taxiing at the Los Rodeos airport runway. The accident investigation report (backed by objective cockpit voice and flight data recorder information) later revealed that human factors contributed to the deadliest mishap in aviation history that claimed 583 lives. Ineffective radio communication with Air Traffic Control due to non-standard terminology and language barrier issues led to misinterpretation of the actual situation and a premature take-off decision of the KLM captain. The steep authority-gradient in the Dutch cockpit prevented the crew from challenging the captain’s decision. As part of the solution to prevent this from happening ever again, a compulsory Human Factor training for all aircrew personnel was advised after follow-up research. This annual training, called Crew Resource Management (CRM), was developed in 1979 in a workshop sponsored by NASA [22]. CRM has meant a huge leap forward in improving aircrew team performance in civilian as well as in military aviation. The focus of CRM is on threat and error management and early identification with blame free countering of human mistakes. By now CRM training is mandatory for professional aircrew in Europe and the USA.

How does this fit in the intensive care environment? In the ICU there is inherently much emphasis on technical skills and not on communication, teamwork and leadership. These latter skills are still rarely deliberately taught or sought after from applicants [21], resulting in critical care practitioners being relatively unprepared to meet the demands of the increasingly complex ICU environment [1,23]. This is the basis for suboptimal coordination of multidisciplinary care and the resultant high number of ICU errors [24]. The traditional critical care environment has a tendency to focus on the performance of a particular practitioner rather than on the system of care.

In aviation, *Non-technical skills*, a *blame-free environment* and *Team Situational Awareness (SA)* are considered CRM core competencies that require specific and focused training [25]. Team SA is defined as the ability to identify, process, and comprehend the critical elements of information about what is happening to the team with regards to the mission. Team SA is considered to be the basis for effective decision making in critical environments and a core competence for any professional team. The archetypical medical specialist’s personality (highly motivated, A-type, control freak) helps in creating an environment in which a junior team member could feel inhibited to offer input in a senior team with “vertical” leadership. This impacts Team SA, posing a threat to process safety, and thus patient safety [23].

### Where is the evidence?

ICUs with a “team-oriented culture” have shorter lengths of stay, lower nursing turnover, higher quality of care and can better meet family members’ needs [26]. As discussed above, general information on a department’s safety climate may be obtained by questionnaires and reviews of complication data [16]. Objective team performance data in regards to specific adverse events is hard to obtain. Although the patient monitor and ventilator store data, the process by which decisions are made is only available afterwards in the form of doctor’s and nurse’s notes. Video monitoring with voice recording is not widely available for debriefing purposes. This limits the visibility of the role of Human Factors in peer reviews and morbidity and mortality conferences. The effect of national rules and regulations cannot be overestimated. In the USA, according to the Health Insurance Portability and Accountability Act (HIPAA), the simple concept of video recording a trauma resuscitation requires review by a lawyer and, according to the Joint Commission in the US, patient permission and is, thus, not a widely accepted practice.

To date no evidence is available from the ICU environment that CRM training improves patient safety. Notably, most team training evaluations have been conducted in the military and aviation environment [27]. These results look promising, and recent evidence also shows a positive effect of team training in the Operating Room [28,29]. Neily et al. analyzed surgical mortality data from 108 Veterans Affairs Hospitals and showed that a Medical Team Training program resulted in significant reduction in surgical mortality rates [29]. Unfortunately, results from other authors are less favourable. Even though non-technical skills, attitudes and teamwork climate seem to improve, no significant effect on operating time, length of hospital stay (LOS) was found [30]. Considerable cultural resistance to adoption is encountered, particularly among medical staff. Debriefing and challenging authority seemed more difficult to adopt than other parts of the training [30].

### Changing the climate: implementation

Crew Resource Management training for Royal Netherlands Air Force (RNLAF) aircrew is a 2-day full time interdisciplinary training. The training syllabus consists of lectures in cognitive psychology and multiple interactive sessions using realistic data.

Key subjects in the CRM-syllabus are:

Situational Awareness and recognition of adverse situations

Human errors and non-punitive response

Communication and crosscheck techniques

Give and receive performance feedback

Management of stress, workload and fatigue

Creating and maintaining team structure and climate

Leadership

Risk management and decision-making

Any CRM-training has to meet Federal Aviation Authority (FAA) or Joint Aviation Authority (JAR) regulations. Not only do they define the various subjects but also the extent to which each subject should be discussed and set limits for refresher training. This standardization is a major contributing factor to the success of CRM.

Medical CRM-training has no international standard yet. Medical Human Factors awareness training initiatives may vary in curriculum, duration, intensity and follow-on support. The U.S. Department of Defense’s Patient Safety Program developed TeamSTEPPS, an evidence-based teamwork system, in collaboration with the Agency for Healthcare Research and Quality (AHRQ). TeamSTEPPS has been implemented in a variety of clinical settings and shown team performance improvement in pediatric and surgical ICUs [31]. Still, any hospital department deciding that CRM is the way forward to improve patient safety should realize it’s not just a single shot training investment (which can be very effective in itself [29]) but part of a culture intervention. There will be understandable reluctance in the medical community to accept the necessity of a CRM-culture intervention in their professional environment. Even though further studies are needed to define the optimal training package [29], some basic guidelines may be given.

1. CRM training. The goal of this training is creating awareness of the human factors that influence team performance. We suggest a 2-day full time training containing the key subjects of aviation CRM as discussed above. As the multidisciplinary ICU environment requires a different non-technical skillset than a cockpit, medical CRM training should be tailored to the specific department’s environment. This is where some current training initiatives fall short. Tailoring CRM-training to the specific needs of an ICU requires insight in the specific clinical processes and culture [32]. ICU professionals have no tradition in briefing and debriefing techniques and performance feedback. ICU-CRM training should therefore emphasize:

briefing and debriefing skills using exercises and actual ICU video footage

the effective use of checklists

identifying team roles

promoting structure, reduction of ineffective communication

performance feedback as an essential requirement in CRM. This starts with careful consideration of timing and relevance of the message, followed by 3 levels of performance feedback. The first level requires the team member to formulate the message short, clear and non-blaming (“doctor, I’m not sure we did all the checks…”). The second level contains a key word that has a defined value (“doctor, I’m not *comfortable* with that decision…”). If this feedback is ignored, and the situation is considered unacceptable the last resort could be a request to “stop the procedure”.

One CRM-tool used at the Mayo Clinic to help facilitate accurate communication in stressful medical situations is based upon the work by Patterson et al [33]. Medical personnel are taught how to communicate without creating conflict or in the face of apparent conflict. This model has proven useful while multiple other endeavors have been created and implemented to foster the ultimate safe environment.

We are currently developing evidence-based requirements for a national ICU-CRM training curriculum in the Netherlands. The basis is a 2-day CRM-training using lectures, video-feedback and interactive exercises. This training is followed by a 1-year implementation phase in which a core group of department professionals is coached by aviation professionals. Results will be published in the near future.

2. CRM implementation. To be successful, the culture change should be supported by additional measures. A core group of ICU-professionals should receive extra coaching during the year after the training to be able to integrate and develop the new way of professional interaction within the ICU-department. And even though CRM relies on intrinsic motivation to be effective, the department leadership needs to clarify to all staff beforehand that CRM is not a noncommittal system but will serve as a yardstick for professional evaluation too. This requires leadership by example.

3. CRM and simulation. The effect of CRM-based culture change is reinforced by the use of scenario-based team training exercises, again derived from aviation simulation expertise [34]. Simulation creates a zero-risk environment that allows medical teams to practice high-risk, low frequency events without endangering patients [25]. This training can either be done in an artificial “laboratory” environment or “in situ”-training, which is conducted on actual patient care units involving actual health care team members and actual organization processes [33]. Simulation – if well debriefed - has many advantages, but if used as a stand-alone modus without the basis of CRM-training holds the risk of focusing too much on technical skills and single-task performance [35]. This will result in a limited impact on patient safety. Key to the success of team training tools in health care is the identification of the domain-specific team skills required for effectively managing routine and emergency scenarios [36]. We suggest to implement two separate phases of simulation training: the first level of training mainly focusing on technical skills, then CRM-training (classroom) followed by second level simulation training that focuses on non-technical performance.

4. CRM retention. Research in military aviation shows that retention of the CRM-subject matter and the effect on aircrew attitude degrades after 3 years. Therefore CRM-refresher training in the RNLAF is scheduled every 3 years [37]. Whether the hospital setting calls for a similar refresher-schedule or regular well-debriefed simulation sessions are effective enough is still unclear.

## Conclusion

Human Factors account for the majority of adverse events in aviation as well as in clinical medicine. The current safety paradigm is still based on ways to limit human variability in otherwise safe systems, promoting stringent procedural guidelines. CRM focuses on improving interprofessional cooperation and team performance and thus patient safety. Even though evidence of CRM on medical errors and patient outcome is still scarce, the parallels between the critical processes in aviation and Intensive Care suggest that a well-adapted medical CRM training has potential for the ICU environment too.

## Abbreviations

AHRQ: Agency for Healthcare Research and Quality (United States); CRM: Crew Resource Management; CUSP: Comprehensive Unit-based Safety Program; FAA: Federal Aviation Authority (United States); HIPAA: Health Insurance Portability and Accountability Act (United States); ICU: Intensive Care Unit; JAR: Joint Aviation Authority (Europe); LOS: Length of Hospital Stay; NASA: North American Space Administration; RNLAF: Royal Netherlands Air Force; SA: Situational Awareness; SAQ: Safety Attitudes Questionnaire.

## Competing interests

All authors declare having no competing interests.

The corresponding author, a board-certified surgeon and retired NL Air Force pilot, has founded the Dutch organisation “Wings of Care” with the aim to implement patient safety measures on a national level.

## Authors’ contributions

MH carried out the main literature search and drafted the manuscript. DJ added relevant literature references and helped shape the article. HH added relevant literature, participated in sequence alignment and provided regular feedback. All authors read and approved the final manuscript.
